# Flexural Properties of Lattices Fabricated with Planar and Curved Layered Fused Filament Fabrication

**DOI:** 10.3390/ma16093451

**Published:** 2023-04-28

**Authors:** José Luis Pérez-Castillo, Angel Mora, Rogelio Perez-Santiago, Armando Roman-Flores, Rafiq Ahmad, Enrique Cuan-Urquizo

**Affiliations:** 1 Tecnologico de Monterrey, School of Engineering and Sciences, Epigmenio González 500 Fracc, San Pablo, Querétaro 76130, Mexico; a01680600@tec.mx (J.L.P.-C.); rogelio.perez@tec.mx (R.P.-S.); 2 Tecnologico de Monterrey, Institute of Advanced Materials for Sustainable Manufacturing, Av. Eugenio Garza Sada 2501, Monterrey 64849, Mexico; armando.roman@tec.mx; 3 Tecnologico de Monterrey, School of Engineering and Sciences, General Ramon Corona 2514, Zapopan 45138, Mexico; 4 Smart & Sustainable Manufacturing Systems Laboratory (SMART LAB), Department of Mechanical Engineering, University of Alberta, Edmonton, AB T6G 2R3, Canada; rafiq.ahmad@ualberta.ca

**Keywords:** curved layer fused filament fabrication, lattice structure, 3D printing, 3-point bending

## Abstract

The use of curved layers in fused filament fabrication could lead to various advantages in surface finishing and mechanical properties. Here, the influence of three different structural and manufacturing parameters (volume fraction, raster arrangement, and the use of curved or planar layers) on the mechanical properties of lattice structures under three-point bending is studied. Two different raster arrangements were considered, i.e., those with rasters at planes parallel to the principal axes of the samples and those diagonally arranged, all at four different volume fractions. All different samples were additively manufactured using planar and curved layers. Samples were further dimensionally inspected to refine the computational models before their analysis via finite element simulations. The linear elastic region of the load-displacement curves was further analyzed numerically via finite element models. Predictions with finite element models resulted in good agreement with errors below 10%. Samples with diagonal rasters were 70% softer than those parallel to the principal axes.

## 1. Introduction

Among the various additive manufacturing (AM) processes and technologies, perhaps the most popular is that of fused filament fabrication (FFF), also known as fused deposition modeling [1,2]. This technology offers many advantages when compared with other additive manufacturing techniques, e.g., a broad range of materials [3,4] and economic accessibility in hardware setup [5]. One area that has benefited the most due to the advent of AM is the field of architected materials. These often use lattice-type arrangements fabricated via AM as they are built by depositing material in a controlled manner [6].

The material arrangement encountered in FFF parts resulting from the controlled deposition has inspired the interested community to characterize their effective mechanical properties [7,8]. Various parameters, both manufacturing and structural, have an impact on the effective mechanical properties. Hence, various works along these lines could be found in the literature for FFF structures [9,10,11]. Popescu et al. [12] concluded that the main parameters affecting the mechanical properties of FFF printed parts are built orientation, raster-to-raster air gap, layer thickness, infill density, and raster angle. It was also demonstrated that optimizing these parameters could enhance surface quality in the FFF parts [10,11].

FFF-lattice and porous structures have many applications that have been studied and documented. For example, Fu et al. studied sound transmission and sound radiation behavior through sandwich structures filled with porous materials [13,14]. Zhou et al. [15] also worked with porous cores in sandwich structures fabricated via FFF, by integrating solid magnesium plates with FFF-lattice cores; namely, the lattices used were based on body-centered cubic topology. Other works that have studied FFF-porous structures in flexural loading scenarios are the works by Cuan-Urquizo and co-workers. For instance, the effective stiffness of polylactic acid-wood [16] and pure polylactic acid (PLA) [17] lattice beams were studied and characterized under three-point bending. The topologies studied in [16,17] could be obtained from conventional FFF slicing software. Other topologies that have also been studied when conforming porous beams are foams [18], chiral-type [19], octet [20], element node [21], and body-centered cubic [22]. A conventional FFF system has three main elements as follows: the printing platform, the filament raw material stock, and the hot end with a nozzle. In general, the function principle of a FFF system consists of two stages: (i) the filament is sucked and semi-melted by the nozzle, and (ii) the nozzle deposits the semi-melted material on the printing bed to build planar layers. The three dimensional (3D) part is then built from the stacking sequence of these layers. FFF parts with out-of-plane curved features built through planar layers result in the so-called stair-case effect, which compromises not only the surface quality of the part but also their mechanical properties.

An alternative form of FFF to overcome the stair-case effect involves using non-planar layers, known as curved layered fused filament fabrication (CLFFF). In CLFFF, the extruder follows out-of-plane trajectories where displacements in the perpendicular axis are simultaneously achieved while moving in any of the other two axes. One advantage of using CLFFF over traditional planar layers is raster continuity in samples with out-of-plane curvature, which results in the enhancement of their mechanical properties. For example, Peng et al. [23] reported that CLFFF samples failed at a higher flexural load than FFF samples, approximately 30.9%. Additionally, in the work of Guang et al. [24], the influence of specific parameters, like filling the gap in flexural strength, was experimentally studied. In [24], the flexural strength of FFF and CLFFF samples was characterized. CLFFF parts presented a higher maximum load capacity of 55% compared to FFF parts. Huang et al. [25] characterized how the raster orientation affects the mechanical behavior of CLFFF parts. In another work, Huang et al. [26] analyzed how the slicing process with different layer thicknesses in samples of the same external dimensions could affect their mechanical properties, obtaining a variation of 6% to 12% between the maximum and minimum number of layers used.

The use of CLFFF in the fabrication of lattice structures is still limited, as most of the works that have evaluated CLFFF’s mechanical properties were focused on quasi-solid samples. Non-planar lattices fabricated via CLFFF have been reported by McCaw and Cuan-Urquizo [6,27], where sinusoid-based parameterized lattices were printed on Bézier surface mandrels and their mechanical properties experimentally evaluated. In both CLFFF and FFF, a detailed characterization of the raster arrangement’s influence on the overall stiffness and strength of the samples is still needed. This is relevant to the production of structures with tailored mechanical properties, as two different topologies made of the same volume fraction(ratio of the volume of material used over the volume they occupied) could result in different effective properties.

Given the gap in the literature exposed, where the use of CLFFF in the fabrication of lattice samples is still an open issue, here we study the mechanical properties of CLFFF-lattice samples under three-point bending and compare them with FFF samples of the same volume fraction. The remaining of this work is structured as follows: Section 2 includes the computational-aided design (CAD) of the CLFFF-lattice samples, their additive manufacturing, and the experimental mechanical setup. Section 3 contains the results from the mechanical properties characterization under three-point bending and their contrast with finite element (FE) estimations. Results are discussed in Section 4. Finally, concluding remarks are presented in Section 5.

## 2. Materials and Methods

**Figure 1:**
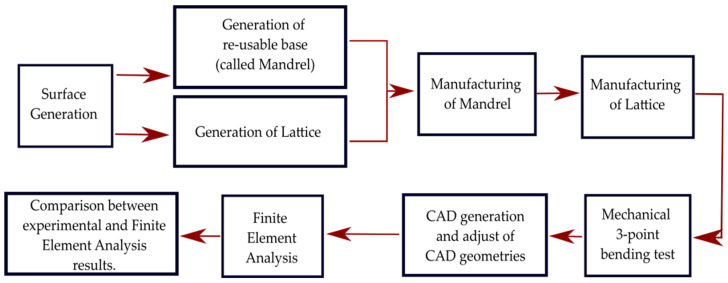
Flowchart of the experiment’s main process.

The general procedure followed in this work comprises the stages shown in the flowchart of Figure 1, which includes the design, computational modeling, and additive manufacturing of lattice samples. Lattice samples with curved external geometry need a target surface that is used for the fabrication of the mandrel and to define the curved lattice sample geometry. Then the lattice samples are mechanically characterized via laboratory and computational experiments.

### 2.1. Desing and Manufacturing of Curved Layered Samples

**Figure 2:**
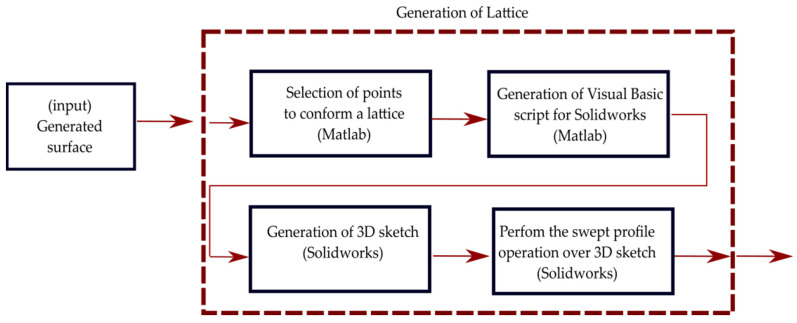
Lattice generation process sub-steps.

The generation, design, and manufacturing of curved lattice samples are achieved through the steps detailed in the following subsections and illustrated in Figure 2.

#### 2.1.1. Target Surface Generation for Mandrel and Lattices

The surface shape that specifies the macro form of the lattice samples is defined first. For this, a MATLAB^®^ script is developed, where a cloud of control points is used as input. The script uses a Bézier-based parametrization for the surface as follows,

(1) $$P\left(u,v\right)=\sum_{j=0}^{m}\sum_{i=0}^{n}P_{i,j}B_{i,n}\left(u\right)B_{j,m}\left(v\right)$$

where u,v∈[0,1] and $P_{i,j}$ are the control points of the surface. The output of the MATLAB^®^ script is a Bézier surface discretized in a cloud of points based on the control points that have been introduced. Since this cloud of points is used in several sections of this work, it is labeled as CLOUD_A. Specifically, here the external geometry of all samples consists of a rectangle of 120 × 30 mm in the XY-plane and variations in the Z-axis of ±2.5 mm.

#### 2.1.2. Mandrel Generation and Additive Manufacturing

**Figure 3:**
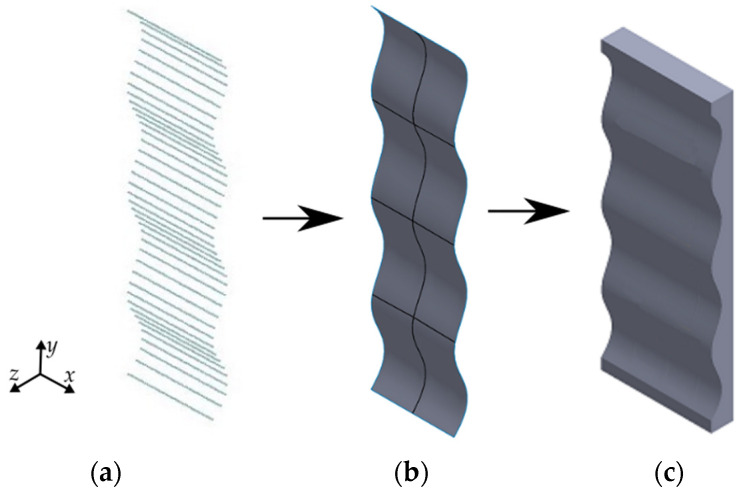
Mandrel generation process: (**a**) cloudpoint, (**b**) mesh surface, and (**c**) extruded mandrel.

CLOUD_A is exported and saved in a .txt file with the format “text delimited by tabs”. Then it is used as input in SolidWorks^®^ (v2019, Dassault Systèmes, Waltham, MA, USA). Within this CAD software, the feature of ScanTo3D is used. This enables importing the CLOUD_A from the .txt file. After the .txt file is uploaded (Figure 3a), a surface mesh is created (Figure 3b). Then, an extrusion operation is used to create a solid model like the one shown in Figure 3c.

The .stl version of the mandrel is further processed in CURA^®^ v4.3.0 (Ultimaker B.V., Utrecht, The Netherlands). The material employed for manufacturing the mandrel and lattices is PLA, with the mechanical properties included in Table 1. The 3D printer used for the mandrel and lattice samples was a Creality^®^ Ender 5 Pro (Longhua Dist., Shenzhen, China). The manufacturing parameters used for the mandrel are listed in Table 2.

**Figure 4:**
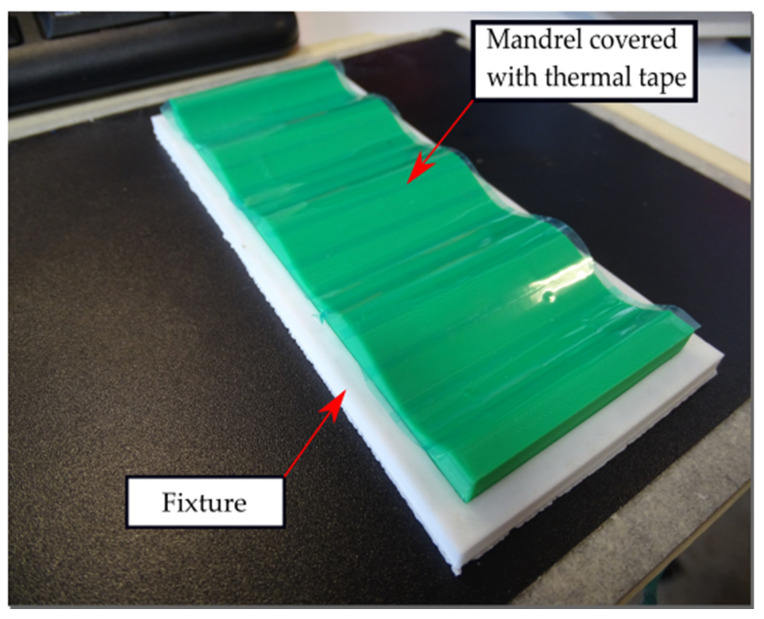
Mandrel and fixture 3D printed setup.

Before the fabrication of the lattice samples, a fixture is printed. This fixture is a rectangular frame that keeps the mandrel in position while other manufacturing operations occur. Since the mandrel and the lattice are printed in the same material and printing parameters, the surface of the mandrel is covered with a thermal tape Mecolour^®^ (sublimation blanks consumables, Shanghai, China) to prevent the lattice adhesion to the mandrel and facilitate the removal process, as shown in Figure 4.

#### 2.1.3. Lattices Generation and Additive Manufacturing of Samples

**Figure 5:**
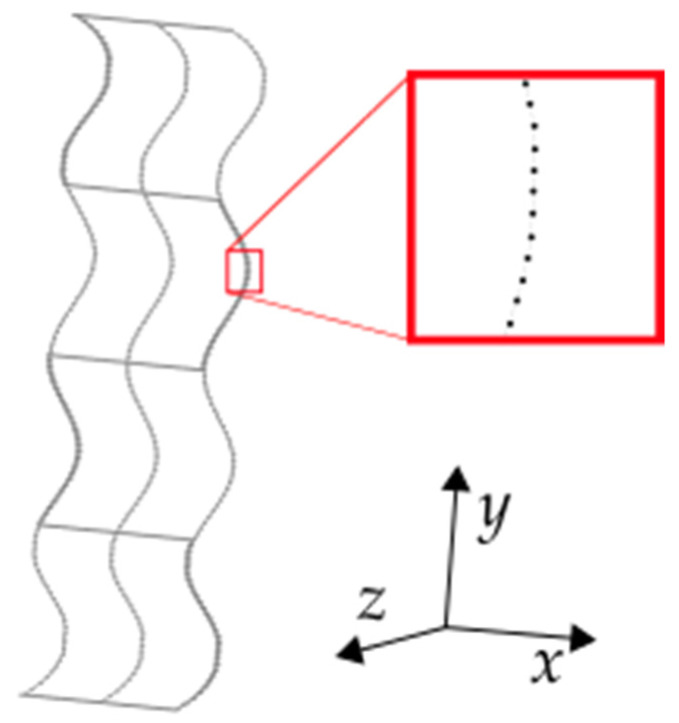
Cloud point labeled as CLOUD_B.

Once CLOUD_A is generated, this is used as input to a second script to conform the lattice in the 3D space. The output of this script is a set of points labeled CLOUD_B. An example of a lattice sample generated with these steps is shown in Figure 5. To manufacture the curved lattice samples, the data in CLOUD_B is arranged so that each row contains the coordinates where the extruder needs to move to build the lattice. These coordinates are then exported in a G-code format, including the manufacturing parameters listed in Table 3 (G0 and G1 are G-code commands used for fast and controlled nozzle displacement, respectively). To achieve non-planar additive manufacturing, here, the nozzle trajectories in the G-code include displacements along the three axes simultaneously. This means that while the nozzle is kept normal to the printing base, its height (location in the Z-axis) varies depending on the shape of the mandrel while moving in the XY-plane. Note that, for this model of 3D printer, the maximum depth or height to avoid collision between the nozzle and mandrel is 5 mm. Five replicas per lattice design were manufactured. The samples were inspected with a magnified photograph to measure their actual dimensions using a microscope Zopsc-1^®^ model 50–1000X. For comparison purposes, lattice samples with equivalent volume fraction as the ones fabricated with CLFFF were also fabricated with flat layers (FFF).

#### 2.1.4. Mechanical Properties Characterization via 3-Point Bending Test

**Figure 6:**
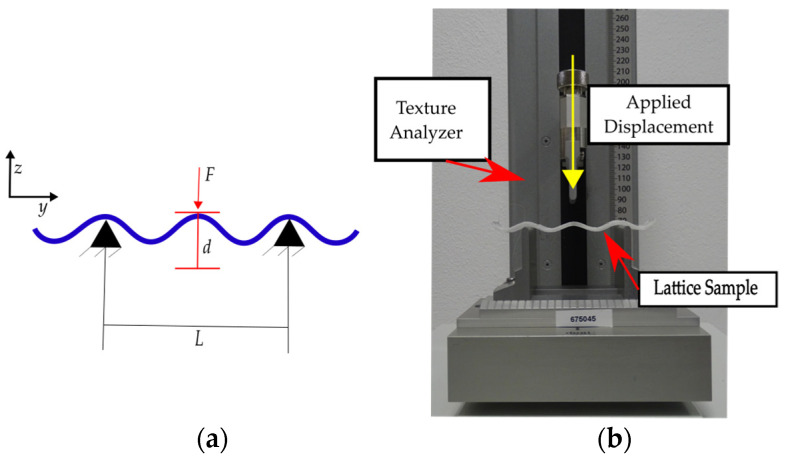
(**a**) Free body diagram of three-point bending test; (**b**) experimental setup.

The mechanical properties of the lattice samples are characterized through three-point bending tests with the boundary conditions shown in Figure 6a (*d* = 30 mm and *L* = 66.5 mm) on a TVT 6700 Perten Texture Analyzer (Perten Instruments, New South Wales, Australia). The machine setup is presented in Figure 6b, showing a curved-lattice sample during testing. The machine was set in displacement control mode setting a maximum displacement of 30 mm at 0.5 mm/s. All variations of the design were tested on five replicas. The data for the load (*F*) and displacement (*d*) was then exported and further processed in MATLAB^®^. All the data for stiffness processed from the three-point bending characterization is included as the mean value along with their corresponding standard deviation.

#### 2.1.5. Curved-Lattice Models and Finite Element Analysis

**Figure 7:**
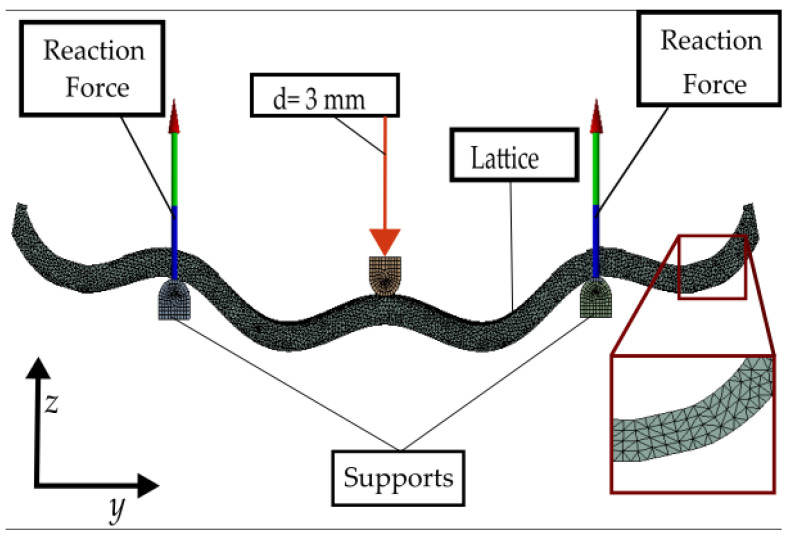
Deformed shape of a lattice in the finite element simulation (reaction force in the image represents the sum of reaction force in both supports).

Cartesian coordinates included in CLOUD_B are used as inputs for a script (macro) made in VisualBasic^®^ Language and run in the SolidWorks^®^ environment. This macro generates a 3D sketch containing all the points at the locations to generate the curved lattice via a swept operation. The computational model developed is then imported to finite element analysis (FEA) software AnsysWorkbench^®^ (Canonsburg, PA, USA), where the applied boundary conditions are shown in Figure 6a. Two types of material properties were used: PLA for lattice models with a Young’s modulus of 3.5 GPa and a Poisson’s ratio of 0.3, and structural steel for supports and loading add-in components, with Young’s modulus of 200 GPa and a Poisson’s ratio of 0.3. The mesh is based on tetrahedral elements with an element size of all bodies (lattice, pusher, and supports) of 1 mm. All simulations are run with a vertical displacement of 3 mm applied, as this displacement is within the linear region of the load-displacement curves in all the experimental measurements. The reaction forces were obtained at the supports. The type of contact between the lattice structure and the supports is frictional with a friction coefficient of 0.2; the type of contact between the pusher and the lattice is bonded because the pusher is in contact with the sample at the same point in every moment of the simulation. Both a deformed shape and the loading conditions in the ANSYS^®^ setup can be observed in Figure 7.

## 3. Results

### 3.1. Manufacturing of Mandrel and Lattices

**Figure 8:**
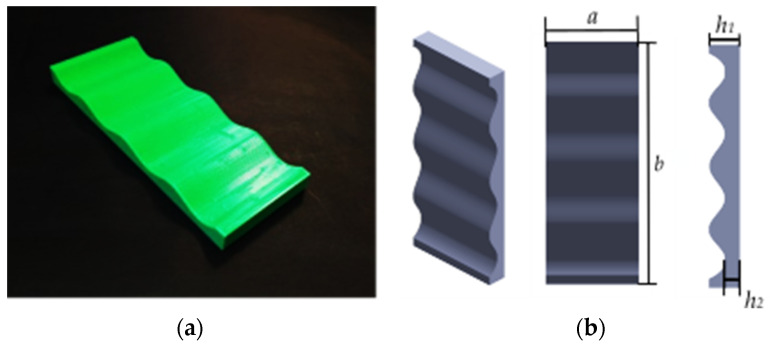
**(a**) Printed mandrel. (**b**) Mandrel dimensions.

**Figure 9:**
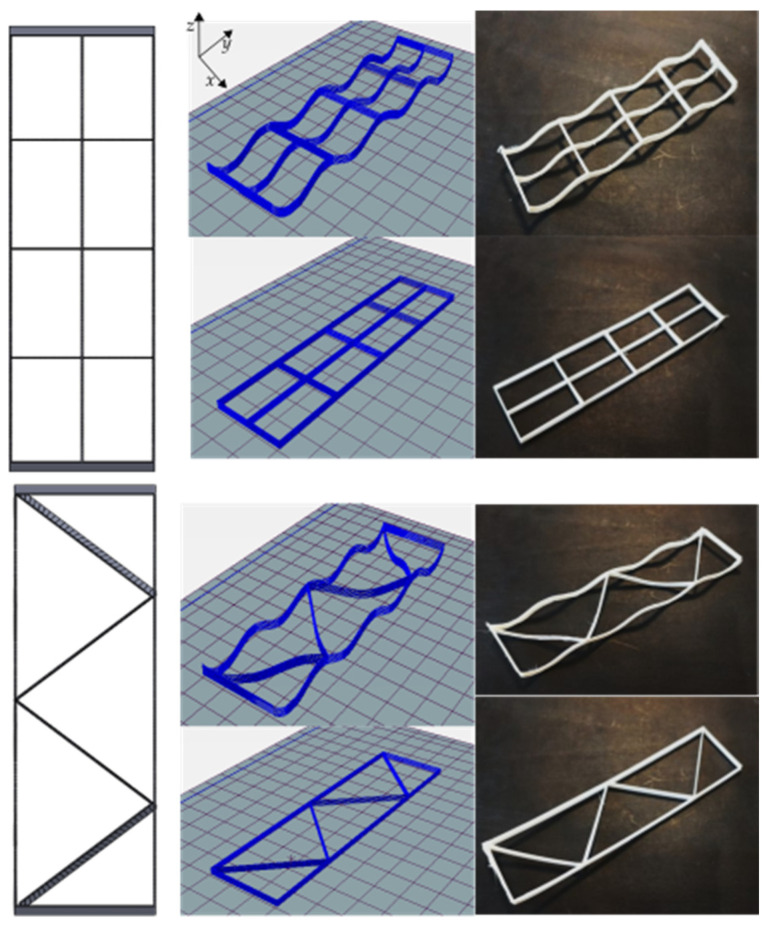
Samples with $\bar{\rho }=$ 0.05 (CAD top view followed by its Repetier^®^ simulation and printed result of two variations, CLFFF and FFF).

**Figure 10:**
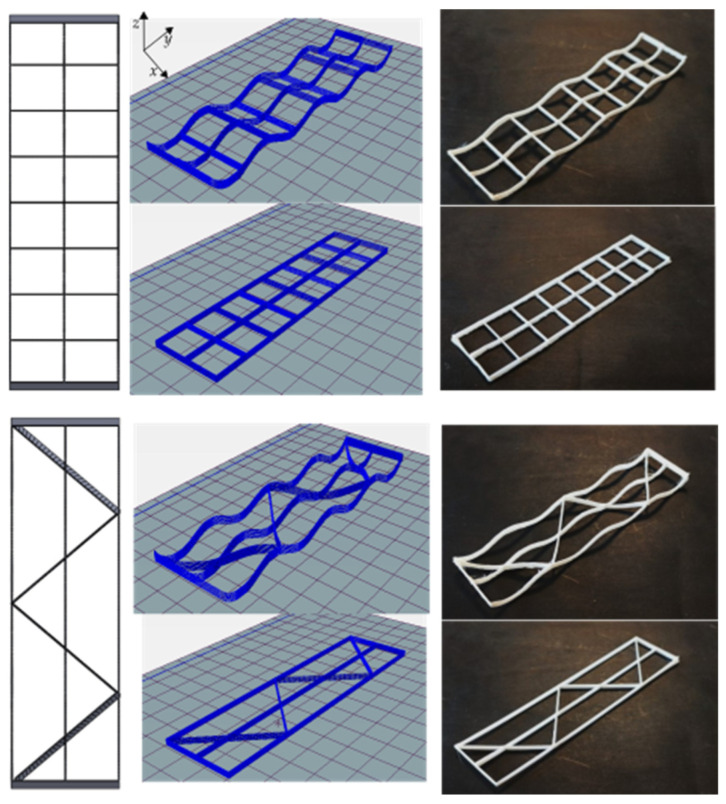
Samples with $\bar{\rho }=$ 0.06 (CAD top view followed by its Repetier^®^ simulation and printed result of two variations, CLFFF and FFF).

**Figure 11:**
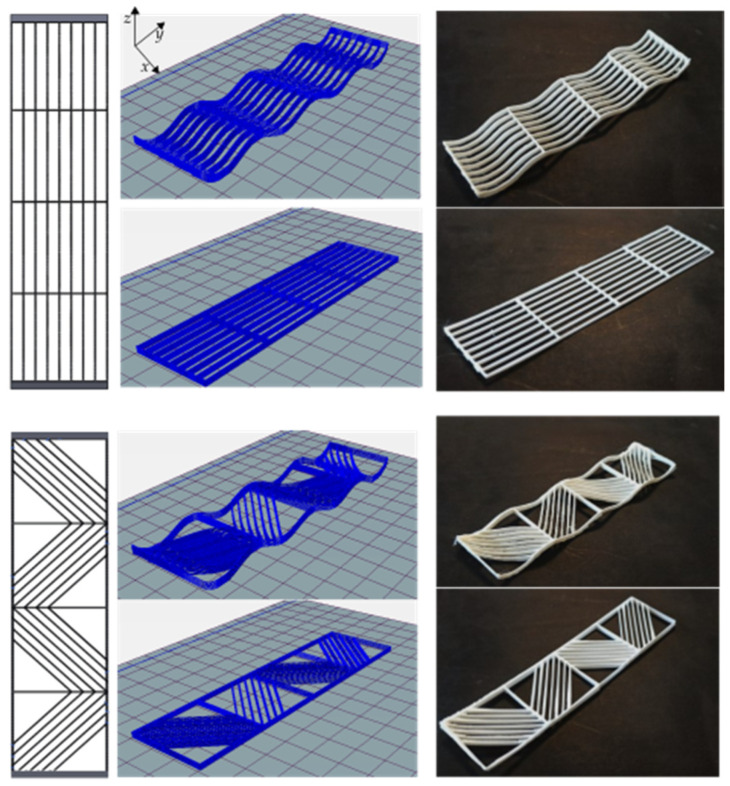
Samples with $\bar{\rho }=$ 0.13 (CAD top view followed by its Repetier^®^ simulation and printed result of two variations, CLFFF and FFF).

**Figure 12:**
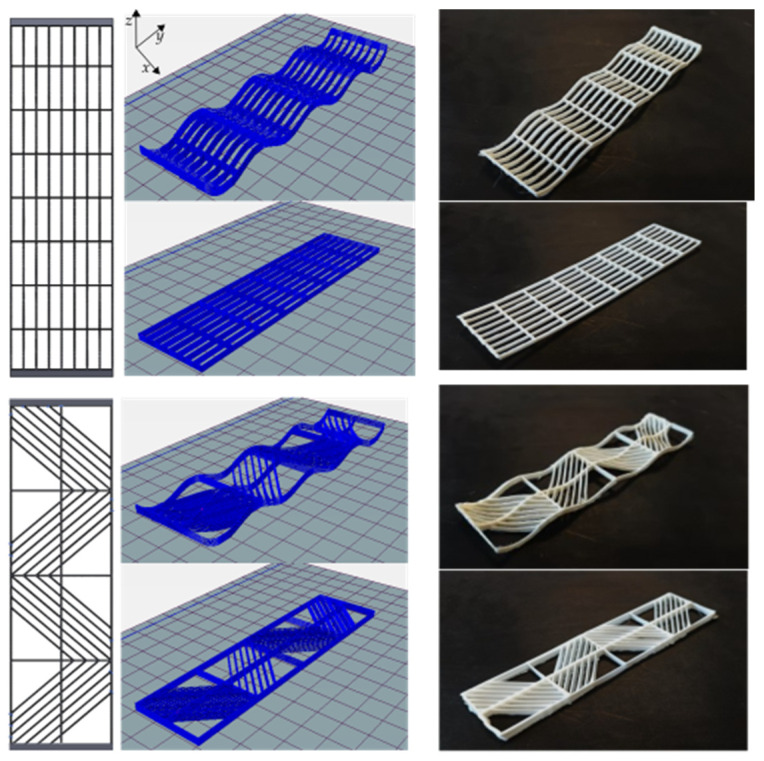
Samples with $\bar{\rho }=$ 0.14 (CAD top view followed by its Repetier^®^ simulation and a printed result of two variations, CLFFF and FFF).

The fabricated mandrel with dimensions of *a* = 50, *b* = 130, *h*_1_ = 10, and *h*_2_ = 5 is shown in Figure 8 (all in millimeters). In CLFFF and FFF, four different volume fractions ($\bar{\rho }$) were printed. For each of these, two raster arrangements were tested (square and diagonal). In Figure 9, Figure 10, Figure 11 and Figure 12, all the lattices designed following the procedure described in Section 2.1 are presented. Samples in Figure 9, Figure 10, Figure 11 and Figure 12 are arranged according to their raster arrangement (square or diagonal), volume fraction, and their fabrication method (CLFFF or FFF). In Figure 9, Figure 10, Figure 11 and Figure 12, from left to right, the CAD model, the extruder trajectory simulation, and the manufactured sample are presented.

**Figure 13:**
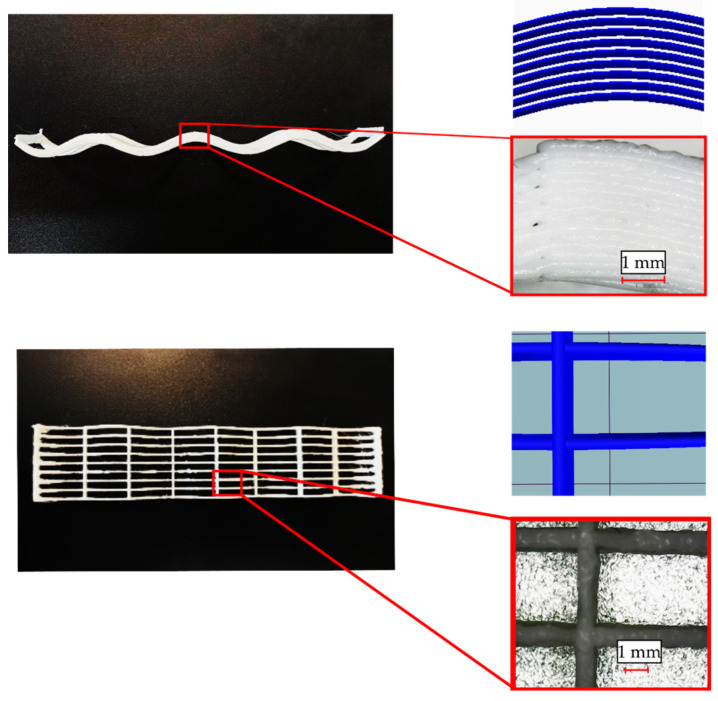
Magnified view of lattice CLFFF structure.

All samples were inspected in a magnified view and compared to the CAD. Expected results in terms of layering and material distribution were observed in the magnified view. This magnified inspection was then used to adjust the dimensions and geometry of the FEA models (Figure 13). Thickness variations in such samples could lead to deviations when compared with the FEA estimations. How these variations could affect the mechanical properties is further discussed in Section 3.2.1. and Section 3.2.2.

### 3.2. Mechanical Properties under 3-Point Bending Loading Scenario

#### 3.2.1. Results of Simulation with CADs of Intended Designed Dimensions

Here the use of finite element analysis is considered for two main motives. First, FEA results are used as estimations of the measured data. Second, FEA opens the opportunity to further explore the deformation mechanism of the lattice samples at the raster level. Stress maps could aid the identification of which rasters have a stronger influence on the effective mechanical properties. There are two scenarios for the results. First (i), the stiffness of lattices with intended design dimensions is analyzed and compared. This initial comparison allows us to evaluate the influence of raster arrangement on the stiffness of the lattice-samples regardless of manufacturing defects. Second (ii), a refinement of the CAD and FE models is done, including the dimensional deviations measured in the additively manufactured samples. These are included in Section 3.2.2.

**Figure 14:**
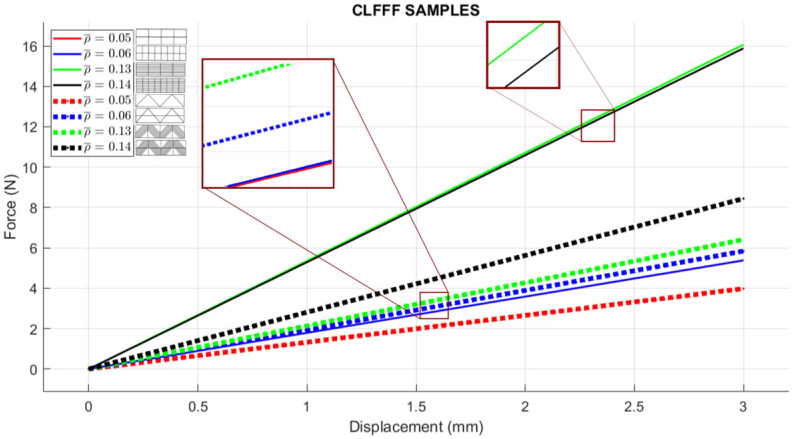
FEA simulation of curved lattices with ideal CAD dimensions (dotted lines represent diagonal patterns; solid lines represent square patterns).

**Figure 15:**
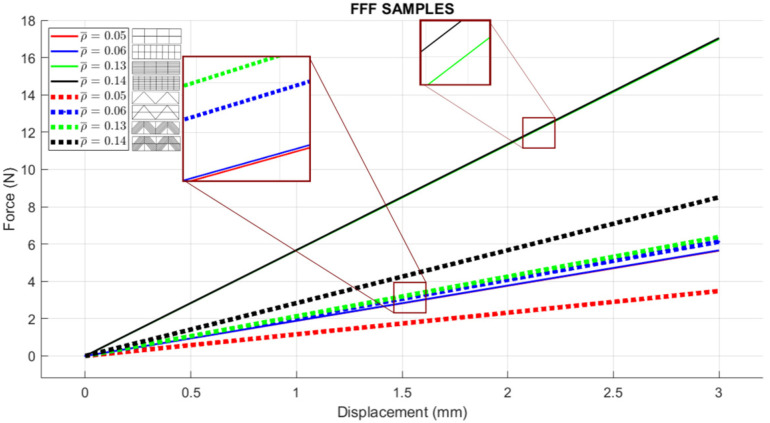
FEA simulation of planar lattices with ideal CAD dimensions (dotted lines represent diagonal patterns; solid lines represent square patterns).

For (i), Figure 14 includes the linear elastic response of the curved lattice samples and Figure 15 for the planar ones, both evaluated via FEA. Dotted lines are used for those samples with diagonal rasters. Note that as the volume fraction increases, structures become stiffer. In general, the so-called square lattices are stiffer than those that include diagonal arrangements. Now, when comparing the stiffness of the square lattice with $\bar{\rho }=0.05$ against those with $\bar{\rho }=0.06$, stiffness values appear to be close to each other (nearly colinear lines in Figure 14). This is attributed entirely to the fact these samples have three axially distributed rasters (see Figure 9 and Figure 10). Diagonal rasters offer less resistance to deformation than the axially distributed, but they do offer more stiffness when compared to those distributed transversally. In Section 4, this observation is further discussed.

#### 3.2.2. Experimental Results and FEA Simulation with CADs with Adjusted Dimensions

**Figure 16:**
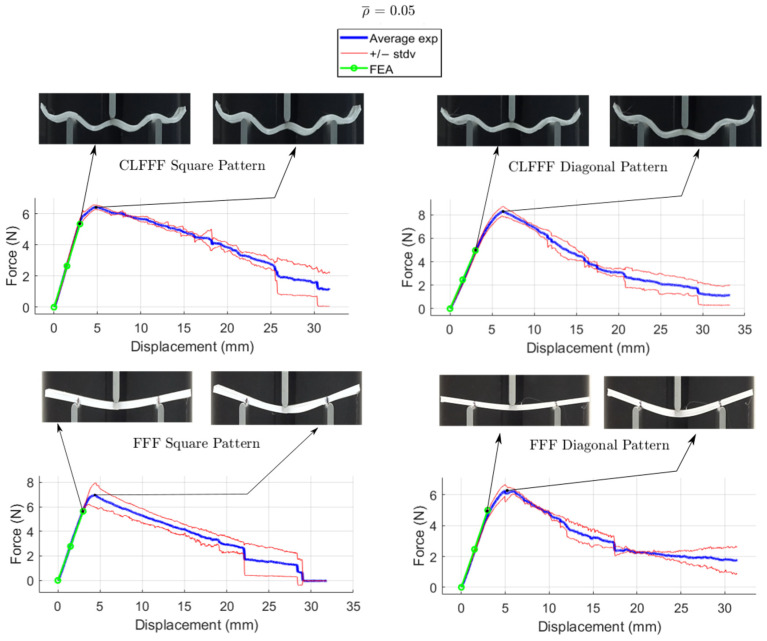
Displacement versus reaction force experiments with $\bar{\rho }=$ 0.05.

**Figure 17:**
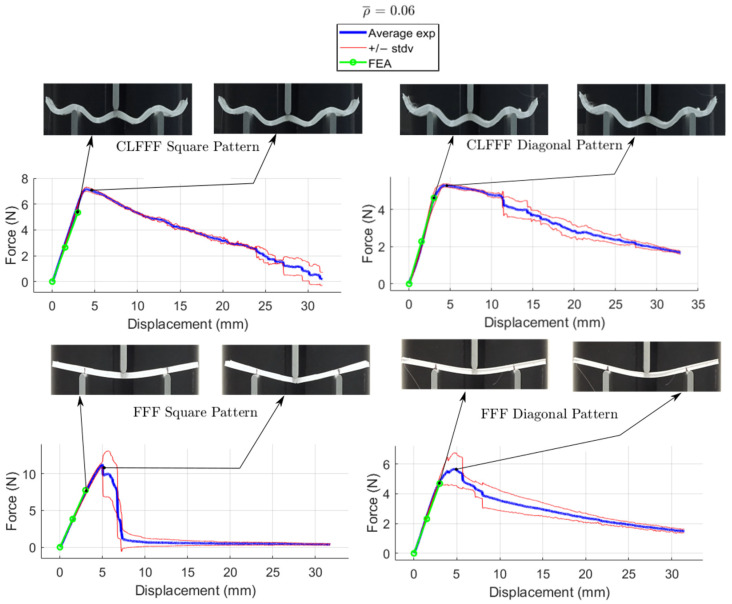
Displacement versus reaction force experiments with $\bar{\rho }=$ 0.06.

**Figure 18:**
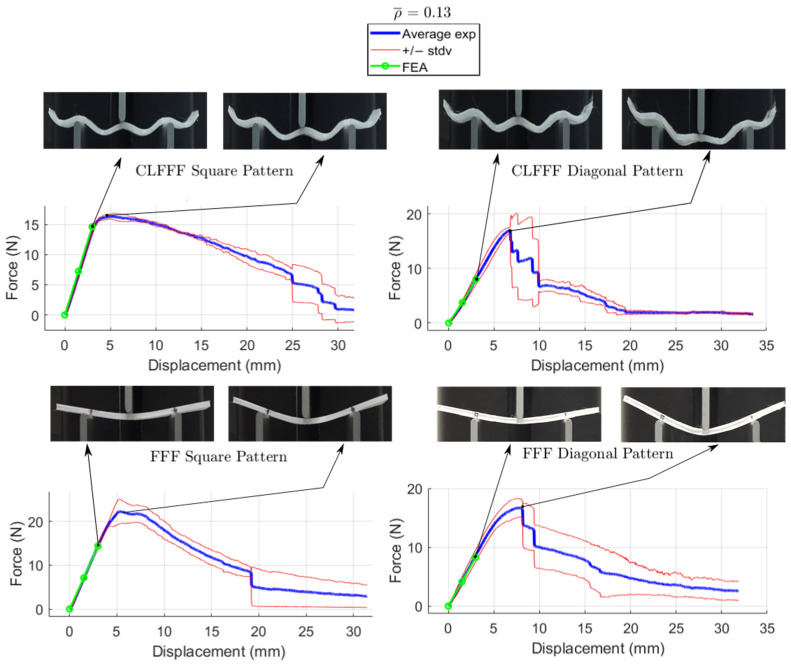
Displacement versus reaction force experiments with $\bar{\rho }=$ 0.13.

**Figure 19:**
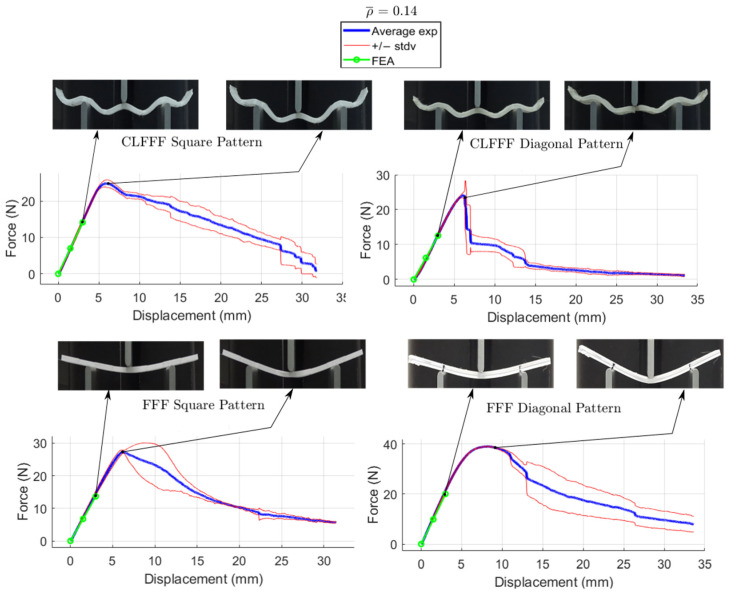
Displacement versus reaction force experiments with $\bar{\rho }=$ 0.14.

The average load-displacement curves of all the tested samples are presented in Figure 16, Figure 17, Figure 18 and Figure 19, where representative photographs of the samples during experiments are also included. Thinner lines in these figures represent the variation of the measurements as these are plotted at +/− standard deviation. Stiffness and maximum peak load measured for each lattice design are summarized in Table 4 and Table 5 for CLFFF and FFF samples, respectively.

**Figure 20:**
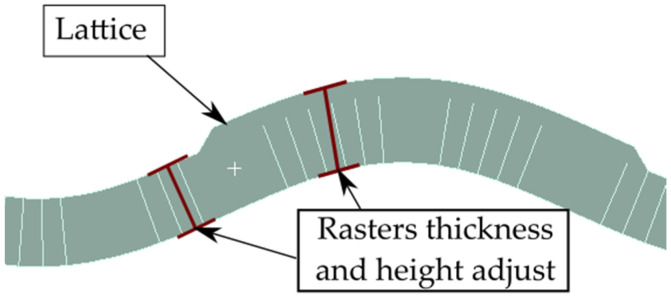
Height and thickness variation in a CLFF sample.

Additively manufactured samples were measured to obtain the actual dimensions for thickness and height. The measurements were used to update the CAD and FEA models (Figure 20). Then, these updated FEA models were subjected to three-point bending, and the stiffness results were compared to those measured from the experimental tests. The stiffness estimation from the FEA models is also included in the plots shown in Figure 16, Figure 17, Figure 18 and Figure 19. Detailed comparisons, along with the errors obtained, are presented in Table 4 and Table 5. In some cases, especially in those with diagonal rasters, CAD models with adjusted dimensions could be in the range of ~40% and ~130% stiffer than those with designed dimensions for CLFFF and FFF, respectively.

#### 3.2.3. Comparison of Results Based on Pattern (Diagonal vs. Square)

For CLFFF, the reaction force, when reaching a displacement of 3 mm, was compared in all experimental measurements. For all volume fractions of CLFFF, the square pattern samples present higher values from 10.9% up to 41.1% compared to diagonal patterns. On the other hand, in FFF samples, only for $\bar{\rho }$ of 0.05, 0.06, and 0.13, the square patterns present higher reaction force values that range from 18.2% up to 40.4%. The only exceptions were the samples with $\bar{\rho }=0.14$, where the diagonal pattern presents a reaction force value 45.9% higher than the square pattern. The latter is attributed to the dimensions deviation obtained in additively manufactured samples when compared to the intended design. This was further corroborated when comparing the stiffness of these samples with ideal dimensions (Figure 14), where the samples that do not include diagonal rasters resulted in being stiffer for all configurations.

#### 3.2.4. Comparison of Results Based on Printed Technology (CLFFF vs. FFF)

For both CLFFF and FFF, the geometries were designed to keep continuity in a material deposition during the printing process, allowing us to compare only the use of out-of-plane curves in conforming the structures. The results presented here correspond entirely to the experimentally obtained data. In general, curved samples are softer than straight ones, as the curved elements in the former tend to be straightened as their initial deformation mechanism. Since straight (FFF) samples are flat in their stress-free state, they are stiffer. For samples with $\bar{\rho }=0.05$, FFF square pattern resulted in being 3.4% stiffer than its corresponding CLFFF sample. Conversely, FFF diagonal samples resulted in being 3.7% more flexible than their CLFFF counterpart. For samples with $\bar{\rho }=0.06,$ the FFF samples resulted in 25.1% and 5.7% higher reaction force than their CLFFF counterparts for the square and diagonal patterns, respectively. For samples with $\bar{\rho }=0.13$ FFF also resulted in higher reaction forces than CLFFF (5.1% in square pattern and 6.4% in diagonal). The highest difference was obtained for samples with $\bar{\rho }=0.14,\;$where lattices with diagonal rasters made with FFF resulted in being 67.1% stiffer than their CLFFF counterparts.

## 4. Discussion

In this work, CLFFF was implemented in a conventional three-axis 3D printer, considering the limitations of the shape and dimension of the nozzle (the maximum depth that this type of nozzle can reach is 5 mm). The first challenge was that manufactured samples presented millimetric variations in their height and thickness. Those millimetric variations drastically affect the mechanical measured properties of each lattice structure. For that reason, the CAD models were adjusted to the actual dimensions of the manufactured samples leading to a refinement of the FE models, obtaining result errors below 10%.

**Figure 21:**
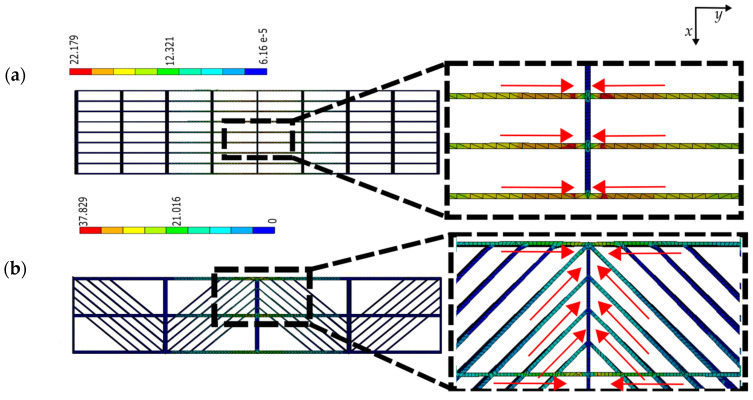
Von Mises stress distribution in (**a**) square and (**b**) diagonal pattern at $\bar{\rho }=$ 0.14 in FFF (units in MPa); red arrows indicate the rasters arrangements with higher influence on the stiffness.

Samples with three different raster arrangements were studied, i.e., axial, transverse, and diagonal. Each contributed differently to the overall stiffness of the lattice samples. Among all, those arranged axially have a stronger influence on the stiffness of the samples. As the samples are subjected to three-point bending, the internal fibers along these axial rasters are subjected to tension and compression, acting along their principal axis. This can be validated by inspecting the stress map distribution in FEA models (Figure 21). Note that the higher magnitudes are in axial rasters, regardless of the presence of transverse or diagonal ones.

**Figure 22:**
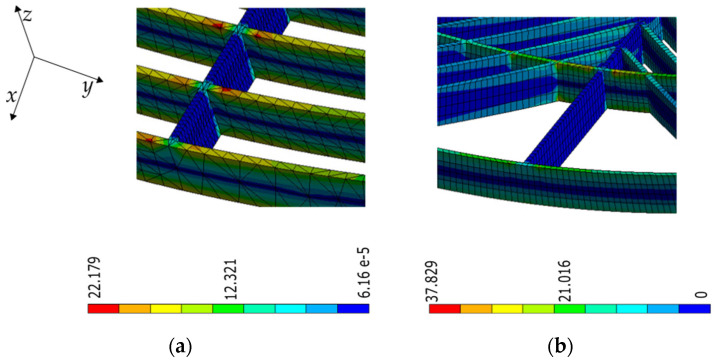
Stress distribution at the raster level resulted from finite element simulations of (**a**) square and (**b**) diagonal lattices in FFF samples (units in MPa).

Transversely arranged rasters encountered in those samples, labeled as “square”, participate minimally in the response. From further inspecting Figure 21a,b, these rasters remain almost stress-free. On the other hand, diagonal rasters do contribute to the overall stiffness of the models (see Figure 21b). The magnitudes of von Misses stress resulting in transverse and diagonal rasters of the FEA simulations were yet analyzed and compared to those axially arranged (Figure 22). Diagonally arranged rasters could result in ~60% of the stress magnitude encountered in the axial ones, compared with the transverse ones, which could result in a maximum of ~3% of stress.

In general, experimental and numerical data exhibited that lattice samples with a higher number of axial rasters are stiffer. Volume fraction also affects the stiffness behavior, but only in cases where axial rasters are included. Take, for instance, the comparison between lattices with diagonal arrangements with $\bar{\rho }=0.05\;\mathrm{and}\;\bar{\rho }=0.06.$ The difference in volume fraction is only 0.01. However, the samples with $\bar{\rho }=0.06$ are ~46% and ~76% stiffer in curved and flat lattices, respectively. Now consider samples with $\bar{\rho }=0.13\;$and $\bar{\rho }=0.14,$ again, the difference in volume fraction is only 0.01. Nevertheless, those structures with $\bar{\rho }=0.14$ are ~30% stiffer. These differences in stiffness are the result of including an axial raster in the lattice structures. Such findings prompt the design of structures with tailored mechanical properties while aiming for a reduction of the amount of material used (minimal volume fraction increment 0.01) and notorious stiffening results (up to ~76% stiffer structures).

In general, curved samples are softer than planar ones. This is attributed entirely to the deformation mechanism that a curved structure offers upon the application of transverse load. When such a loading scenario is encountered, the curved elements tend to be straightened first, which is not occurring in the flat samples; hence, the latter are stiffer.

## 5. Conclusions

The mechanical properties under the three-point bending of additively manufactured lattices were experimentally and numerically characterized. Two FFF approaches were explored, i.e., the traditional with planar layers and that of CLFFF. The influences of the two main parameters were studied, i.e., the volume fraction and the rasters arrangement. The experimental and numerical data presented here demonstrated that two samples with the same volume fraction could result in very different stiffness properties due to the raster arrangements. Axially arranged rasters have a higher withstanding of the flexural load applied. Then when comparing diagonal and transverse rasters, the latter participate minimally in the overall stiffness of the samples. These series of conclusions are a key factor in the design (and fabrication) of components with desired mechanical properties while taking into consideration the amount of matter employed in their building.

### Future Work

The results presented in this work demonstrated the relevance of raster arrangements in the mechanical properties of CLFFF and FFF structures. These prompt further research that needs to be pursued. For example, all the CLFFF samples presented here were fabricated with conventional FFF machines, i.e., three degrees of freedom (DOFs). Fabricating structures with more complex geometries could benefit from the inclusion of additional DOFs in the hardware of the additive manufacturing machines. Other lattice arrangements could result in interesting mechanical properties, for example, those with curvature at the raster level (sinusoid lattice). Finally, other loading scenarios need to be studied in addition to the conventional tension/compression and bending, e.g., torsional or cyclic loads.

## Figures and Tables

**Table 1:** Mechanical properties of polylactic acid (PLA).

Property	Value
Heat Deflection Temperature (HDT)	49–52 °C at 0.46 MPa
Tensile Strength	61–66 MPa
Flexural Strength	48–110 MPa
Young’s Modulus	3.5 Gpa
Poisson’s Ratio	0.3

**Table 2:** Mandrel printing parameters.

Parameter	Value
Bed temperature	60 °C
Infill	10%
Built Plate Adhesion	Raft
Material	Generic PLA
Nozzle Diameter	0.4 mm
Hot end temperature	205 °C
Printing Speed	70 mm/s
Layer Height	0.2 mm

**Table 3:** Lattice printing parameters.

Parameter	Value
Bed temperature	25 °C
Hot end temperature	210 °C
Feed rate (G0)	3000 mm/s
Feed Rate (G1)	375 mm/s

**Table 4:** Comparison of texture analyzer results against finite element analysis in lattices printed via CLFFF.

$\mathrm{Volume}\;\mathrm{Fraction}\;(\bar{\rho })$	Pattern	Stiffness (N/mm) Mean ± Standard Deviation	Error between Experimental Result and FEA
0.05	Square	1.894 ± 0.193	2.8%
	Diagonal	1.643 ± 0.256	2.5%
0.06	Square	2.014 ± 0.058	9.9%
	Diagonal	1.576 ± 0.358	1.5%
0.13	Square	4.73 ± 0.291	6.7%
	Diagonal	2.74 ± 0.997	0.5%
0.14	Square	4.938 ± 0.561	1.7%
	Diagonal	4.451 ± 0.252	2.0%

**Table 5:** Comparison of texture analyzer results against finite element analysis in lattices printed via FFF.

$\mathrm{Volume}\;\mathrm{Fraction}\;(\bar{\rho })$	Pattern	Stiffness (N/mm) Mean ± Standard Deviation	Error between Experimental Result and FEA
0.05	Square	1.953 ± 0.090	1.3%
	Diagonal	1.608 ± 0.213	7.2%
0.06	Square	2.522 ± 0.353	4.3%
	Diagonal	1.683 ± 0.317	2.3%
0.13	Square	4.976 ± 0.444	0.1%
	Diagonal	2.909 ± 1.023	3.4%
0.14	Square	4.983 ± 0.686	6.8%
	Diagonal	7.286 ± 0.294	6.4%

## Data Availability

Data is contained within the article.
